# Delays in Appropriate Antibiotic Therapy for Gram-Negative Bloodstream Infections: A Multicenter, Community Hospital Study

**DOI:** 10.1371/journal.pone.0076225

**Published:** 2013-10-03

**Authors:** Rebekah W. Moehring, Richard Sloane, Luke F. Chen, Emily C. Smathers, Kenneth E. Schmader, Vance G. Fowler, David J. Weber, Daniel J. Sexton, Deverick J. Anderson

**Affiliations:** 1 Department of Medicine, Division of Infectious Diseases, Duke University Medical Center, Durham, North Carolina, United States of America; 2 Division of Geriatrics, Duke University Medical Center, Durham, North Carolina, United States of America; 3 Geriatric Research Education and Clinical Center, Durham Veterans Affairs Medical Center, Durham, North Carolina, United States of America; 4 Department of Medicine, Division of Infectious Diseases, Durham Veterans Affairs Medical Center, Durham, North Carolina, United States of America; 5 Department of Epidemiology, University of North Carolina at Chapel Hill, Chapel Hill, North Carolina, United States of America; Arizona State University, United States of America

## Abstract

**Background:**

Gram-negative bacterial bloodstream infection (BSI) is a serious condition with estimated 30% mortality. Clinical outcomes for patients with severe infections improve when antibiotics are appropriately chosen and given early. The objective of this study was to estimate the association of prior healthcare exposure on time to appropriate antibiotic therapy in patients with gram-negative BSI.

**Method:**

We performed a multicenter cohort study of adult, hospitalized patients with gram-negative BSI using time to event analysis in nine community hospitals from 2003-2006. Event time was defined as the first administration of an antibiotic with *in* *vitro* activity against the infecting organism. Healthcare exposure status was categorized as community-acquired, healthcare-associated, or hospital-acquired. Time to appropriate therapy among groups of patients with differing healthcare exposure status was assessed using Kaplan-Meier analyses and multivariate Cox proportional hazards models.

**Results:**

The cohort included 578 patients with gram-negative BSI, including 320 (55%) healthcare-associated, 217 (38%) community-acquired, and 41 (7%) hospital-acquired infections. 529 (92%) patients received an appropriate antibiotic during their hospitalization. Time to appropriate therapy was significantly different among the groups of healthcare exposure status (log-rank p=0.02). Time to first antibiotic administration regardless of drug appropriateness was not different between groups (p=0.3). The unadjusted hazard ratios (HR) (95% confidence interval) were 0.80 (0.65-0.98) for healthcare-associated and 0.72 (0.63-0.82) for hospital-acquired, relative to patients with community-acquired BSI. In multivariable analysis, interaction was found between the main effect and baseline Charlson comorbidity index. When Charlson index was 3, adjusted HRs were 0.66 (0.48-0.92) for healthcare-associated and 0.57 (0.44-0.75) for hospital-acquired, relative to patients with community-acquired infections.

**Conclusions:**

Patients with healthcare-associated or hospital-acquired BSI experienced delays in receipt of appropriate antibiotics for gram-negative BSI compared to patients with community-acquired BSI. This difference was not due to delayed initiation of antibiotic therapy, but due to the inappropriate choice of antibiotic.

## Introduction

Timely and appropriate antimicrobial therapy is critically important for treatment of patients with severe infections. Inadequate timing or choice of antibiotics increases the risk that patients will die; even a few hours of delay increases mortality risk in patients with severe infections [1-7]. Multiple studies examined the detrimental effects of inappropriate empiric antibiotic therapy on outcomes of death, length of stay, and hospital costs [1,4,6,8,9]. A meta-analysis of >70 studies, including non-intensive care populations, demonstrated that inappropriate empiric antibiotic therapy was associated with a 1.6-fold increase in mortality [1]. These findings have led to dedicated efforts to minimize the time to administration of appropriate antibiotics [10].

Although the impact of inappropriate empiric antibiotic therapy has been well-established, unanswered questions remain. Specifically, what types of patients are at risk for delayed appropriate therapy? Antibiotic management of Gram-negative infections is increasingly difficult due to drug resistance and lagging antibiotic development [11]. Gram-negative bloodstream infections carry a mortality rate of 30% or worse when inappropriate antibiotics are given [7,12]. Risk factors for delayed appropriate therapy in patients with Gram-negative bloodstream BSI are not fully understood. Finally, the majority of US healthcare is delivered in community hospitals; however antibiotic prescribing practices are rarely studied in this practice setting [13].

Prior studies have identified patient groups at increased risk for inappropriate empiric antibiotic therapy, which include patients with Gram-negative infections or nosocomial acquisition [2,4,14-18]. However, prior studies have not critically examined the time to administration of appropriate antibiotic therapy. Prior investigators used a pre-defined time point (e.g. 24 hours after blood culture collection) to create a binary measure of inappropriate empiric antibiotic therapy. Researchers focused on a short window in time to capture empiric choices, rather than addressing the larger goal of administering effective therapy as early as possible. Consequently, we understand little about how antibiotic treatment decisions evolve over time in the course of a patient’s illness.

Time to appropriate antibiotic therapy has recently been proposed as a quality measure and needs further study [19]. This process-based outcome could be used to evaluate the quality of antimicrobial management or antimicrobial stewardship programs [19].

The objective of this multicenter cohort study was to estimate the effect of healthcare exposure status on the time to receive appropriate antibiotic therapy in patients with Gram-negative BSI from the community hospital setting.

### Ethics Statement

This study was reviewed and approved by the Institutional Review Board (IRB) of Duke University Health System. Participating community hospitals deferred to the Duke IRB (n=5), or reviewed and approved the study via their local IRB (n=4). Written patient consent was waived by all sites.

## Methods

This retrospective cohort study included patients at nine community, non-academic hospitals in North Carolina and Virginia affiliated with the Duke Infection Control Outreach Network (DICON) [20,21]. The median size of participating hospitals was 151 (range 102-355) beds.

BSI events were defined using Centers for Disease Control and Prevention (CDC) criteria for laboratory-confirmed BSI: ≥ 1 positive blood culture for all bacterial pathogens except common skin contaminants, which required ≥ 2 positive blood cultures within a 48 hour period [22,23]. The BSI definition was applied to all blood culture results in participating hospitals’ microbiology databases from January 1, 2003 to December 31, 2006. Data abstractors reviewed charts randomly selected by a computer-generated list and applied two eligibility criteria: age ≥18 years old, and events occurring during hospitalization or within one day prior to admission. Data from 1,478 patients with bloodstream infections were entered into the cohort database. Clinical, microbiologic, and treatment data were collected from 24 hours prior to admission through discharge. Patients with Gram-negative BSI pathogens were selected from the larger cohort for the current study.

### Variable Definitions

Time of BSI event was defined as collection date and time of first positive blood culture growing the Gram-negative pathogen. The main outcome was time to receipt of appropriate antibiotic therapy.

Antibiotic therapy was appropriate if two criteria were met: 1) the pathogen was susceptible to the antibiotic *in vitro*, and 2) antibiotic and route of administration would provide adequate bioavailability for treatment of BSI. For example, a pathogen may show *in vitro* susceptibility to nitrofurantoin; however this was deemed inappropriate for BSI due to poor antimicrobial activity in plasma.

Time to appropriate therapy was defined as starting 24 hours prior to collection of first positive blood culture (“time 0”) and ending with receipt of first dose of appropriate antibiotic (the event). Ideally, time 0 starts with the first opportunity for a clinician to recognize the onset of bacteremia; however, this was impossible to measure retrospectively. Therefore, we included appropriate antibiotic choices made during the 24 hours leading up to collection of blood cultures. Although blood cultures should be collected prior to initiation of antibiotic therapy, empiric antibiotic therapy before obtaining blood cultures is a common practice. To be clear, time 0 was defined as the time of blood culture collection minus 24 hours for the purposes of this analysis. Thus, the time interval analyzed in this study included time in the emergency room for patients presenting from the community. Patients who were receiving an appropriate antibiotic for >24 hours at the time of blood culture collection were defined as “breakthrough” BSIs [24]. Breakthrough BSIs were excluded from analysis because patients had experienced the event prior to start of the risk period.

Polymicrobial infection was defined as presence of >1 pathogen during any single BSI event. If multiple Gram-negative pathogens were present during the same BSI event, appropriate therapy did not occur until at least one agent active against each Gram-negative pathogen was administered. Patients who never received an appropriate antibiotic were administratively censored at noon on day of discharge or 240 hours (10 days), whichever came first. Patients who received an appropriate antibiotic >10 days from time 0 were censored at 240 hours and considered not to have experienced the outcome. Administrative censoring was utilized to dampen the skewing effect of the few patients who had a prolonged admission without appropriate therapy.

Time to any antibiotic therapy, regardless of appropriateness, was explored as a secondary outcome and defined similarly. Time 0 started 24 hours prior to collection of first positive blood culture. Event was receipt of first dose of any antibiotic without consideration of *in vitro* activity or bioavailability. Administrative censoring was the same as described above.

Healthcare exposure status was the main exposure of interest, categorized into three groups using a definition modified from Friedman et al. [25] Healthcare-associated BSI occurred < 48 hours from admission and patients had ≥1 of the following healthcare exposure risk factors: 1) presence of an invasive device or 2) history of prior hospitalization, surgery, dialysis, or residence in long-term care facility within one year preceding the BSI event. Community-acquired BSI occurred < 48 hours after admission in patients without healthcare exposure risk factors. Hospital-onset BSI occurred ≥ 48 hours after hospital admission.

Multidrug-resistant (MDR) phenotype was defined using published consensus definitions [26]. Infection source was defined based on microbiologic and clinical data. If the same pathogen was isolated from blood and another body site, the non-blood site was presumed to be the source. If a patient had a central venous catheter and no matching culture from another body site, the source was presumed to be the vascular catheter.

Charlson comorbidity index measured at hospital admission and APACHE II score measured on day of BSI were calculated as previously described [27,28].

### Statistical Analysis

Descriptive statistics summarized the patient cohort. Kaplan-Meier curves were constructed for time to appropriate therapy and time to any antibiotic therapy. Log-rank tests were used to compare among groups of healthcare exposure status. Alpha level of 0.05 and two-sided tests were used with any exceptions noted below.

Cox proportional hazard models were fit to measure the hazard ratios (HR) and 95% confidence intervals (CI) for the association between healthcare exposure and time to appropriate therapy. Proportional hazards assumptions were tested using time-dependent variables. Clustering due to hospital-specific factors were accounted for using the methods of Lee et al. and the robust covariance estimator [29]. Patients with community-acquired BSI were the referent category. Indicator variables for healthcare-associated and hospital-acquired BSI provided separate comparisons to the referent.

Covariates were chosen from factors theorized to impact the association between healthcare exposure and time to appropriate therapy. Covariates directly used in definitions of healthcare exposure (e.g., presence of an invasive device) were excluded. Charlson index and age were determined *a priori* to be important and were included in modeling regardless of statistical significance.

Selected covariates were evaluated for effect measure modification using hierarchical models with interaction terms and likelihood ratio tests (LRT) for model fit (*a priori* alpha<0.1). Each covariate deemed not to be a potential modifier was evaluated individually for association with the main exposure (*a priori* alpha<0.05) among all patients, and then the main outcome among patients in the referent category (*a priori* alpha<0.1) to determine potential confounding.

Covariates significant in analyses of interaction or confounding were included in the initial multivariate model and then evaluated for removal using a backward elimination approach. Covariates were removed for the final model based on two criteria: 1. Model terms for interaction did not improve model fit by LRT<0.1; 2. Model terms for possible confounders did not affect the absolute change in HR >10% among any strata of effect measure modifier or improve model fit by LRT<0.05 when compared to the initial model.

Two separate multivariate models were constructed. Model 1 was designed to estimate the full association of healthcare exposure status and did not adjust for intermediate factors that occur as a result of healthcare exposure: organism type, polymicrobial infection, MDR, source of infection, and severity of illness. Intermediate variables were adjusted for, however, in design of an exploratory Model 2 to estimate the direct association between healthcare exposure and time to appropriate therapy.

All analyses were completed using SAS Version 9.3.1 (Cary, North Carolina).

## Results

### Description of Study Cohort

The study cohort included 578 patients with Gram-negative BSIs after removal of 19 patients with breakthrough BSI. A total of 320 (55%) patients had healthcare-associated, 217 (38%) had community-acquired, and 41 (7%) had hospital-acquired BSI (Table 1). Most patients were elderly (median age 72 [range 18-97]). The majority were on Medicare or Medicaid (81%). Over half (56%) had impairment in at least one activity of daily living.

**Table 1:** Characteristics of Patients by Healthcare Exposure Status.

			**Total Cohort (N =578)^a^**	**Community-acquired (n=217)^a^**	**Healthcare-associated (n=320)^a^**	**Hospital-acquired (n=41)^a^**	**P value^e^**
**Patient Characteristics**	Age, years, median (range)		72 (18-97)	69 (18-97)	73.5 (19-97)	73 (36-94)	0.01
	Male Sex		257 (44)	85 (39)	146 (46)	26 (63)	0.01
	Non-white Race		266 (47)	87 (40)	164 (52)	15 (34)	0.02
	Medicare/Medicaid		463 (81)	158 (74)	267 (85)	38 (83)	<.001
	No insurance		31 (5)	18 (8)	12 (4)	1 (2)	0.06
	BMI, median (range)		25 (13-55)	27 (13-55)	24 (13-55)	24 (18-54)	0.004
	Charlson score,^b^median (range)		3 (0-11)	1 (0-11)	3 (0-10)	3 (0-9)	<.001
	APACHE II,^c^median (range)		14 (4-30)	13 (4-28)	15 (4-30)	14 (7-29)	0.006
	MCCabe score ^b^						<.001
		Rapidly fatal <2 weeks	105 (19)	35 (17)	56 (18)	14 (35)	
		Ultimately fatal <5 years	302 (52)	82 (39)	199 (62)	21 (51)	
		Nonfatal	159 (28)	95 (45)	59 (18)	5 (12)	
	Dependent in ≥1 ADL ^b^		323 (56)	91 (42)	208 (65)	24 (59)	<.001
	Dependent in ≥3 ADL ^b^		136 (24)	24 (11)	107 (34)	5 (11)	<.001
	Admission source ^b^	Home	440 (75)	211 (100)	201 (64)	28 (61)	^d^
		Skilled nursing facility	121 (21)	0 (0)	114 (36)	7 (15)	
		Other hospital	6 (1)	0 (0)	0 (0)	6 (13)	
	Prior hospitalization in last 1 year ^b^		251 (43)	0 (0)	232 (73)	19 (41)	^d^
	Comorbidities ^b^	Diabetes	220 (38)	70 (32)	147 (46)	8 (17)	<.001
		MI	118 (20)	40 (18)	63 (20)	15 (33)	0.03
		CHF	109 (19)	29 (13)	68 (21)	12 (26)	0.02
		PVD	77 (13)	26 (12)	44 (14)	7 (15)	0.6
		Stroke	112 (19)	21 (10)	86 (27)	5 (5)	<.001
		Dementia	102 (18)	21 (10)	78 (24)	3 (7)	<.001
		COPD	100 (17)	31 (14)	58 (18)	11 (24)	0.1
		Liver disease	36 (6)	15 (7)	17 (5)	4 (9)	0.6
		Dialysis	35 (6)	0 (0)	32 (10)	3 (7)	^d^
		Active Malignancy	127 (22)	36 (17)	80 (25)	11 (24)	0.05
		Metastatic Malignancy	26 (4)	6 (3)	16 (5)	4 (9)	0.09
		Decubitus ulcer	79 (14)	11 (5)	62 (19)	6 (13)	<.001
		Immunosuppression	56 (10)	21 (10)	31 (10)	4 (9)	0.99
	Devices ^b^	Central IV catheter	61 (10)	0 (0)	47 (15)	14 (30)	^d^
		Foley catheter	64 (11)	0 (0)	61 (19)	3 (7)	^d^
		PEG	28 (5)	0 (0)	23 (7)	5 (11)	^d^
**Infection Characteristics**	Polymicrobial		48 (8)	10 (5)	30 (9)	8 (17)	0.004
	Multiple GN pathogens		18 (3)	4 (2)	10 (3)	4 (9)	0.1
	Multidrug Resistance		84 (15)	20 (9)	58 (18)	6 (13)	0.02
	Primary Source						0.008
		Urine	96 (17)	42 (19)	48 (15)	6 (15)	
		Wound	4 (1)	2 (1)	2 (1)	0 (0)	
		Lower respiratory tract	8 (1)	2 (1)	3 (1)	3 (7)	
		Other	9 (2)	3 (1)	2 (1)	4 (10)	
		No culture or unknown	406 (70)	168 (77)	223 (70)	15 (37)	
	IV catheter related		55 (10)	0 (0)	42 (13)	13 (32)	<.001
**Outcomes**	Time to appropriate antibiotic therapy, hours, median (95% CI)		26.9	25.9	27.9	27.3	0.02
			(26.2, 28.3)	(25.4, 27.7)	(26.6, 28.9)	(25.3, 48.0)	
	Time to any antibiotic therapy, hours, median (95% CI)		26.4	25.8	27.2	26.4	0.3
			(25.8, 27.5)	(25.2, 26.8)	(26.1 28.2)	(25.0, 36.8)	
	Disposition	Death or hospice	82 (14)	17 (8)	53 (17)	12 (29)	<.001
		Discharge to skilled nursing facility	193 (34)	18 (8)	102 (32)	9 (22)	<.001
		Discharge home with home health services	42 (7)	10 (5)	28 (9)	4 (10)	0.2
	ICU admission		151 (26)	65 (30)	70 (22)	16 (40)	0.02
	Intubation		55 (10)	19 (9)	27 (9)	9 (22)	0.02
	Vasopressors		66 (12)	26 (12)	32 (10)	8 (21)	0.3
	Readmission in 90 days		143 (25)	42 (20)	93 (29)	8 (20)	<.001
	Length of stay post BSI, days, median (range)		6 (0-158)	5 (0-158)	6 (0-143)	7 (0-52)	0.3

Note: Data are number (%) of bloodstream infection events unless otherwise indicated.

BMI = body mass index; APACHE II = Acute Physiology and Chronic Health Evaluation II score; ADL = activity of daily living; MI = myocardial infarction; CHF = congestive heart failure; PVD = peripheral vascular disease; COPD = chronic obstructive pulmonary disease; IV = intravenous; PEG = percutaneous endoscopic gastrostomy; GN = gram negative; CI = confidence interval; ICU = intensive care unit; BSI = bloodstream infection.

a Sums may not equal total N due to small numbers of missing data (not shown) which were less than 10% for any single variable and distributed evenly among groups of healthcare exposure.

b Measured on admission.

c Measured on day of bloodstream infection.

d Significance testing not performed on variables directly used in definitions of healthcare exposure groups.

e P values indicate chi-squared or Fisher exact tests for categorical variables, or ANOVA or Kruskall-Wallis tests for continuous variables, as appropriate. All tests were two-sided, using two degrees of freedom. Time to appropriate therapy and time to first therapy were assessed using log-rank tests.

A total of 598 Gram-negative pathogens were isolated from 578 patients (Table 2). Forty-eight (8%) BSIs were polymicrobial; 18 (3%) involved more than one Gram-negative pathogen. The predominant pathogens were *Enterobacteriaceae*. *E. coli* was observed more commonly in community-acquired BSI, whereas *Enterobacter* was predominately found in hospital-acquired BSI.

**Table 2:** Distribution of Gram-negative Bloodstream Infection Pathogens.

	**Total Cohort (N =598)^a^**	**Community-associated (n=221)**	**Healthcare-associated (n=331)**	**Hospital-acquired (n=46)**
***E. coli***	330 (55)	148 (67)	167 (50)	15 (33)
***Klebsiella***	91 (15)	42 (19)	42 (13)	7 (15)
***Proteus***	53 (9)	7 (3)	40 (12)	6 (13)
***Pseudomonas***	43 (7)	9 (4)	31 (9)	3 (7)
***Enterobacter***	30 (5)	3 (1)	15 (5)	12 (26)
***Acinetobacter***	10 (2)	1 (0)	9 (3)	0 (0)
***Citrobacter***	9 (2)	1 (0)	7 (2)	1 (0)
***Serratia***	9 (2)	3 (1)	6 (2)	0 (0)
***Salmonella***	7 (1)	2 (1)	5 (2)	0 (0)
**Other^b^**	16 (3)	5 (2)	9 (3)	2 (4)

Note: Data are number (%) of isolates unless otherwise indicated.

a 598 pathogens were isolated from 578 patients. 18 patients had >1 Gram-negative pathogen isolated.

b Other organism types include *Morganella, Providencia, Stenotrophomonas, Pasteurella, Alcaligenes*. All had frequencies of <5 (1%) of bloodstream infection events.

MDR phenotype was observed in 84 (15%) patients in the entire cohort. MDR was more common in healthcare-associated (n=58, 18%) and hospital-acquired (n=6, 13%) BSIs, than in community-acquired BSIs (n=10, 9%) (Table 1). Seventy percent of patients had no identified source for their BSI. The remaining patients had a matching culture from an alternate body site (n=117, 20%) or were presumed to have a central line-associated infection (n=55, 10%).

A total of 1,648 courses of antibiotics were administered to study patients during their admission (mean 2.2, standard deviation 1.3 per patient). The most common antibiotics administered were ceftriaxone (n=294, 18%), levofloxacin (n=207, 13%), ciprofloxacin (n=189, 12%), and piperacillin-tazobactam (n=149, 9%).

We observed significant differences in secondary outcomes, including mortality, discharge to skilled nursing facility, intubation, and readmission in 90 days among the three groups of healthcare exposure status (Table 1).

### Kaplan-Meier Analysis

**Figure 1:**
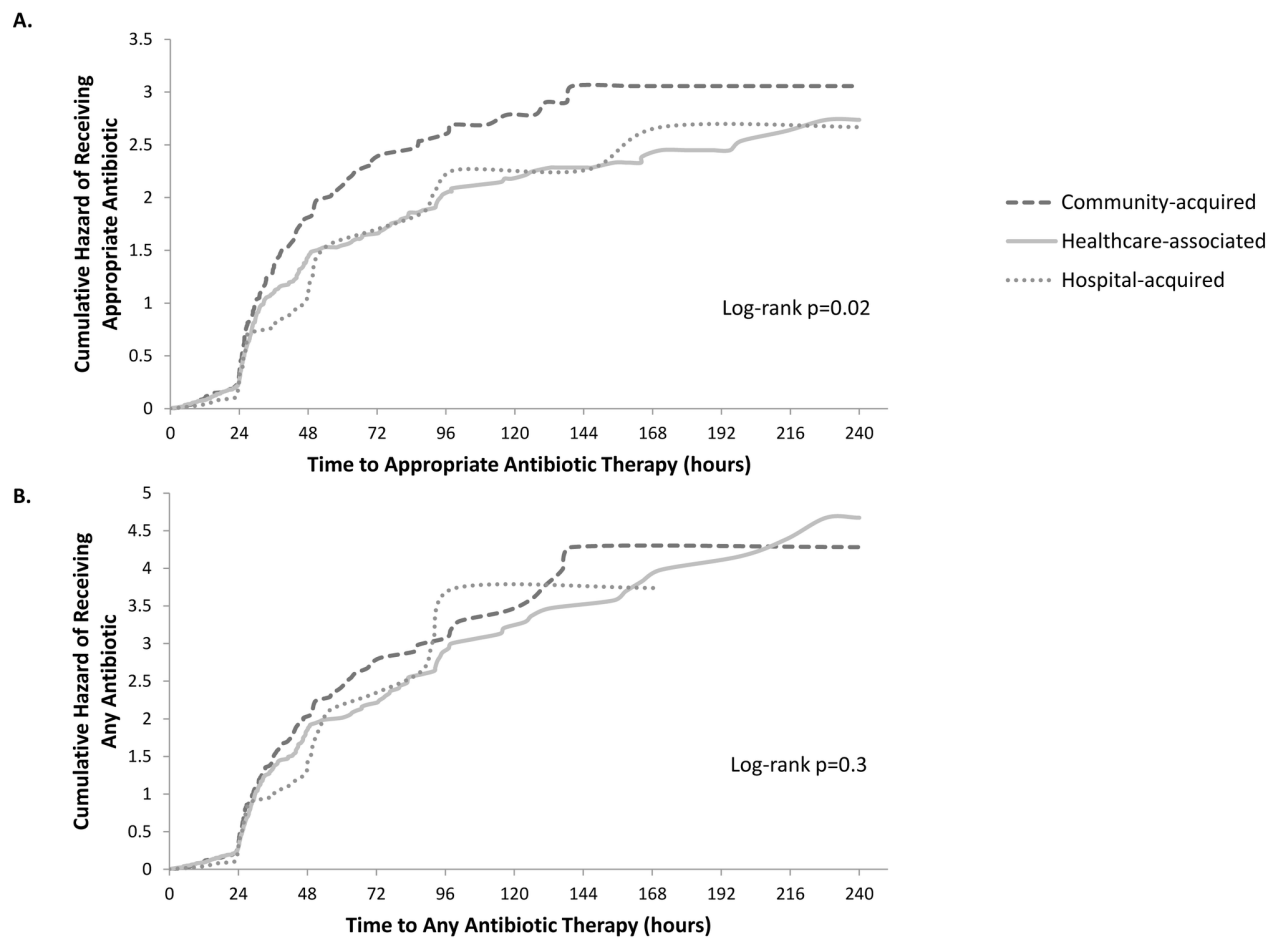
Cumulative hazard of receiving appropriate antibiotic therapy or any antibiotic therapy based on Kaplan-Meier analysis. Cumulative hazards among healthcare exposure categories of patients with Gram-negative bloodstream infections in community hospitals, 2003-2006. Healthcare exposure categories include community-acquired (dashed dark-gray line), healthcare-associated (solid light-gray line), and hospital-acquired (dotted light-gray line). For all analyses, the origin of the risk period was defined as starting 24 hours prior to blood culture collection. A. The cumulative hazard of receiving the first dose of an appropriate antibiotic, log-rank test p=0.02. B. The cumulative hazard of receiving the first dose of any antibiotic, log-rank test p=0.3.

Five hundred twenty-nine (92%) of 578 patients received appropriate therapy during the 10-day risk period. Forty-nine (8%) patients were administratively censored at discharge or day 10. Time to appropriate therapy was significantly different for the three groups (log-rank p=0.02, Figure 1A). Unadjusted analysis revealed median (95% CI) time to appropriate therapy of 25.9 (25.4, 27.7) hours among community-acquired, 27.9 (26.7, 28.9) hours among healthcare-associated, and 27.3 (25.3, 48.0) hours among hospital-acquired patients. Larger differences in time to appropriate therapy for each healthcare exposure group were seen after the first 24 hour time period or for the latter two quartiles of patients. For example, 75% of patients with community-acquired infections had received an appropriate antibiotic by 36.6 (32.9-43.4) hours. In contrast, it took 47.4 (42.8-63.6) hours for 75% of the healthcare-associated, and 51.3 (43.6-99.5) hours for 75% of the hospital-acquired groups to receive appropriate therapy.

All study patients received at least one antibiotic in the 10 days following BSI. The time to any antibiotic therapy, regardless of effectiveness, was not significantly different among the three groups of healthcare exposure status (log-rank p=0.3) (Figure 1B).

### Multivariate Analysis

Unadjusted Cox analysis suggested that patients with healthcare-associated (HR 0.80, 95% CI 0.65-0.98, p=0.03) or hospital-acquired (HR 0.72, 95% CI 0.63-0.82, p=<.001) BSI experienced delays in receiving appropriate therapy when compared to patients with community-acquired BSI. Tests for violation of the proportional hazards assumption were not significant.

Charlson score, body mass index, and active malignancy were found to be potential effect measure modifiers. We pursued a single interaction only due to lack of power to evaluate multivariable interactions. Therefore, only interaction terms between Charlson and the main exposure were included in subsequent modeling.

**Figure 2:**
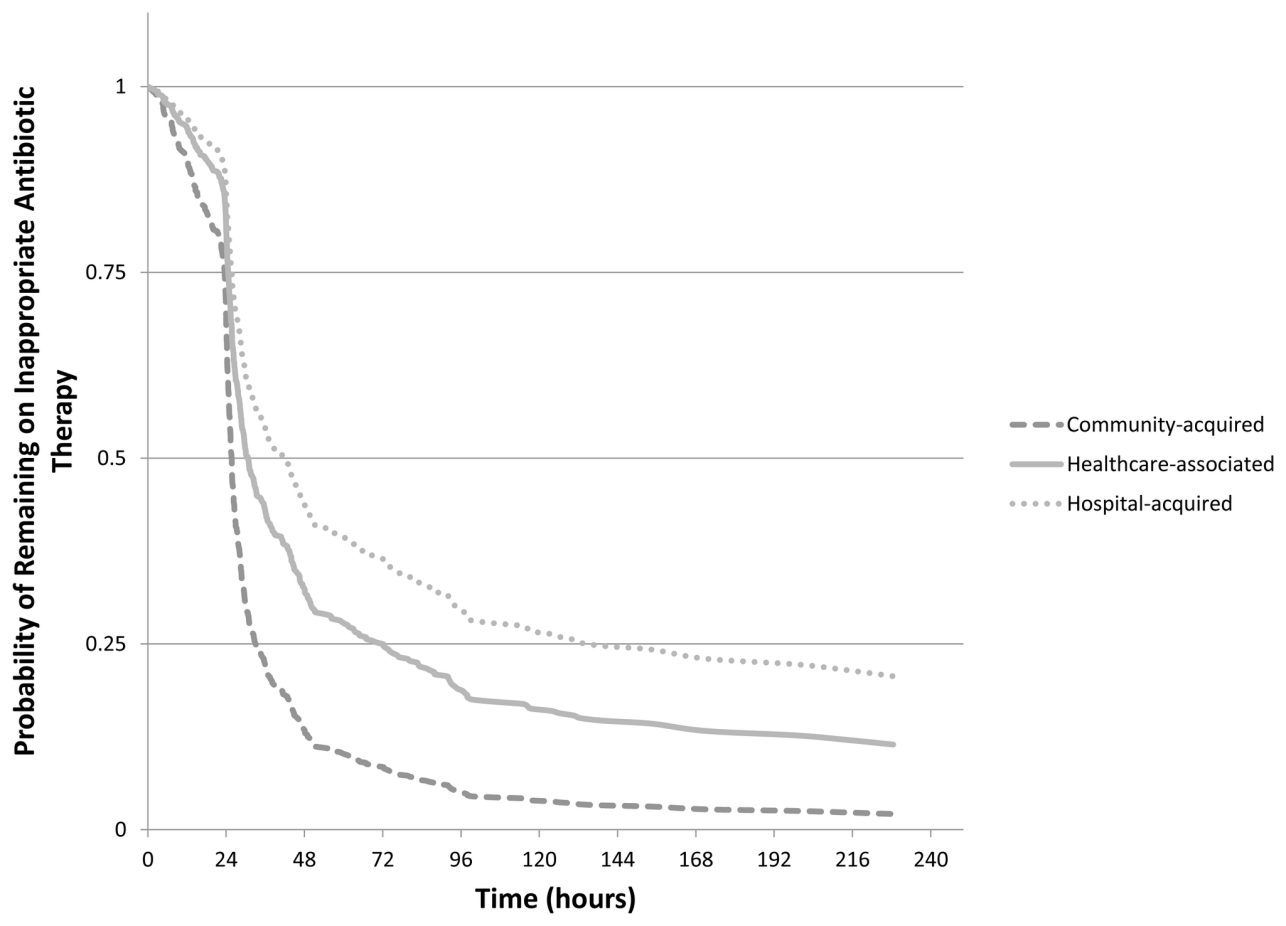
Probability of remaining on inappropriate antibiotic therapy based on Cox proportional hazards model. Healthcare exposure categories include community-acquired (dashed dark-gray line), healthcare-associated (solid light-gray line), and hospital-acquired (dotted light-gray line). Model inputs to produce these curves were the following: Charlson index of 0, malignancy=none, age >65, Medicare/Medicaid=yes, dependent in >3 activities of daily living=yes.

Exclusion of variables based on *a priori* criteria reduced the covariate pool to Charlson, age, body mass index, male sex, Medicare/Medicaid, active malignancy, non-white race, and functional status. Following backward elimination, the final adjusted Model 1 included the main exposure, Charlson plus interaction terms, age, active malignancy, Medicare/Medicaid, and dependence in ≥ 3 activities of daily living. Final adjusted HR (aHR) estimates among strata of Charlson revealed that healthcare-associated and hospital-onset infections were associated with delay in receipt of appropriate antibiotic therapy compared with community-acquired infections (Table 3, Model 1, Figure 2). The effects were strongest among patients with lower Charlson index and hospital-acquired BSI (aHR 0.41, 95% CI 0.26-0.65). The aHR became weaker and included the null in the strata of higher Charlson index among patients with hospital-acquired BSI (aHR 0.90, 95% CI 0.46-1.77).

**Table 3:** Effect of Healthcare Exposure on Time to Appropriate Antibiotic Therapy.

**Charlson score**	**Healthcare Exposure Status**	**Adjusted Hazard Ratio (95% CI)**	
		Model 1^a^	Model 2^b^
**Charlson score=0**	Community-acquired	1	1
	Healthcare-associated	0.56 (0.33-0.95)	0.60 (0.34-1.05)
	Hospital-acquired	0.41 (0.26-0.65)	0.51 (0.32-0.80)
**Charlson score=3**	Community-acquired	1	1
	Healthcare-associated	0.66 (0.48-0.92)	0.64 (0.46-0.89)
	Hospital-acquired	0.57 (0.44-0.75)	0.65 (0.49-0.85)
**Charlson score=7**	Community-acquired	1	1
	Healthcare-associated	0.82 (0.70-0.98)	0.71 (0.60-0.85)
	Hospital-acquired	0.90 (0.46-1.77)	0.90 (0.50-1.62)

Note: 566 patients were included in the multivariate analyses due to small amounts (2%) of missing data distributed among the covariates used for adjustment.

a Adjusted for the following covariates: Charlson index plus interaction terms, age, Medicare/Medicaid, Dependent in >3 activities of daily living

b Adjusted for the following covariates: Charlson index plus interaction terms, age, Medicare/Medicaid, Dependent in >=3 activities of daily living, polymicrobial infection, organism type (monomicrobial infections with *E. coli, Klebsiella*, Non-fermenter, other Enterobacteriaceae, other); multidrug-resistance; primary source type; APACHE II score at the time of bloodstream infection.

Model 2 also included adjustment for intermediate causal factors: organism type, polymicrobial infection, MDR, source of infection, and APACHE II score. Individual stratums’ aHR estimates generally remained significantly different from, but slightly closer to, the null when compared to Model 1 (Table 3, Model 2). In other words, patients with healthcare exposures demonstrated delayed time to appropriate therapy despite adjustment for potentially explanatory factors like MDR, polymicrobial infection, organism type, and source of infection.

## Discussion

This multicenter study from the community hospital setting demonstrated that patients with healthcare-associated or hospital-acquired Gram-negative BSI experienced delay in receipt of appropriate antibiotics when compared to patients with community-acquired BSI. Studies that previously examined the effect of healthcare exposures on inappropriate empiric antibiotic therapy generally did not use a time to event analysis [4,8,14,15,18,30,31]. Also, the patient cohort included community hospital patients with Gram-negative BSI which is an understudied population in existing research examining inappropriate antibiotic therapy.

The time to appropriate therapy was significantly different among community-acquired, healthcare-associated, and hospital-acquired patients in unadjusted analysis. In contrast, time to receipt of the first dose of any antibiotic, regardless of appropriateness, was not significantly different among the patient groups. This suggests that reasons for the delay were not due to the logistics of administering antibiotics, failure to recognize a need for antibiotics, or decisions not to treat, but due to selection of an inappropriate antibiotic. The association persisted in multivariate analyses despite adjustment for confounders as well as explanatory intermediate factors, like MDR and organism type. Thus, a higher rate of drug-resistance only partially explains our observation of delayed appropriate therapy in healthcare-exposed patients.

We theorize that a higher degree of clinical uncertainty involved in evaluation of patients with healthcare exposures may explain these findings. When choosing empiric therapy, clinicians must assess an individual’s epidemiologic risk factors and search for a primary infection. However, practicing clinicians are often faced with complicated, confusing, and sometimes mysterious clinical presentations. For example, hospitalized patients may develop acute complications during their inpatient stay (e.g., drug reaction, delirium) that make clinical evaluation more challenging. These healthcare-related events were not captured in our study.

Time of initial clinical evaluation may also impact the degree of clinical uncertainty. Community-acquired patients who present for care several days after infection onset may have clear manifestations of their primary source. In contrast, healthcare-exposed patients may be evaluated early in their infection course and lack symptoms of a localized source. Early access to care could affect disease latency at the time of first clinical evaluation and thus impact the ease of diagnosis and antibiotic choice.

We found evidence of interaction with baseline comorbidity and the association between healthcare exposure and time to appropriate therapy. The hazard ratio appeared strongest when Charlson index was low, which may be related to lack of statistical power when Charlson was high. Alternatively, more appropriate (or aggressive) antibiotic therapy may have occurred in patients with high baseline comorbidity. We cannot, however, make this conclusion with our data.

Definition of the time component for appropriate antibiotic therapy is not standardized. Most prior studies have examined inappropriate empiric therapy, defined as the lack of receipt of an effective antibiotic within 24 hours from blood culture collection, or in the 48 hour period surrounding blood culture collection (e.g. 1 day before to 1 day after) [3,18,32]. In the few studies that have used time to appropriate therapy, the time period prior to blood culture collection was generally not discussed [32-34]. These studies were also limited to specific pathogens. Using Kaplan-Meier analysis, we discretely observed how appropriate antibiotics were administered from 24 hours before blood culture collection up to 10 days later (Figure 1). Importantly, 25% of patients received appropriate therapy before collection of blood cultures. Exclusion of this group in previous studies may have resulted in over-representation of patients with delayed therapy. Further, this suboptimal culture collection practice impairs ascertainment of bacteremic patients.

This study is limited by its retrospective design, which may have resulted in measurement error or uncontrolled confounding. Limited data could be gathered regarding a primary source of BSI, and the true timing of blood culture collection as compared to the documented date and time could not be validated. Also, unmeasured or unknown confounding factors could have affected the multivariate analysis. Second, the study period 2003-2006 may not reflect current antibiotic prescribing practices or prevalence of multidrug resistance. Third, we did not examine antibiotic dose, which is important for optimal antibiotic therapy. We believe, however, that this study has relevance for today’s prescribers. It highlights the need to assess healthcare-associated risk factors and provides greater understanding of effects of prior healthcare exposure on treatment decisions.

Early, effective antibiotic therapy is important for providing high quality care for patients with Gram-negative BSI. We demonstrated that healthcare-exposed patients are at risk for delayed receipt of appropriate antibiotics.
